# Kinin B_1_ Receptor Enhances the Oxidative Stress in a Rat Model of Insulin Resistance: Outcome in Hypertension, Allodynia and Metabolic Complications

**DOI:** 10.1371/journal.pone.0012622

**Published:** 2010-09-07

**Authors:** Jenny Pena Dias, Sébastien Talbot, Jacques Sénécal, Pierre Carayon, Réjean Couture

**Affiliations:** 1 Department of Physiology, Faculty of Medicine, Université de Montréal, Montréal, Québec, Canada; 2 Sanofi-Aventis R&D, Montpellier, France; Louisiana State University, United States of America

## Abstract

**Background:**

Kinin B_1_ receptor (B_1_R) is induced by the oxidative stress in models of diabetes mellitus. This study aims at determining whether B_1_R activation could perpetuate the oxidative stress which leads to diabetic complications.

**Methods and Findings:**

Young Sprague-Dawley rats were fed with 10% D-Glucose or tap water (controls) for 8–12 weeks. A selective B_1_R antagonist (SSR240612) was administered acutely (3–30 mg/kg) or daily for a period of 7 days (10 mg/kg) and the impact was measured on systolic blood pressure, allodynia, protein and/or mRNA B_1_R expression, aortic superoxide anion (O_2_^•−^) production and expression of superoxide dismutase (MnSOD) and catalase. SSR240612 reduced dose-dependently (3–30 mg/kg) high blood pressure in 12-week glucose-fed rats, but had no effect in controls. Eight-week glucose-fed rats exhibited insulin resistance (HOMA index), hypertension, tactile and cold allodynia and significant increases of plasma levels of glucose and insulin. This was associated with higher aortic levels of O_2_^•−^, NADPH oxidase activity, MnSOD and catalase expression. All these abnormalities including B_1_R overexpression (spinal cord, aorta, liver and gastrocnemius muscle) were normalized by the prolonged treatment with SSR240612. The production of O_2_^•−^ in the aorta of glucose-fed rats was also measured in the presence and absence of inhibitors (10–100 µM) of NADPH oxidase (apocynin), xanthine oxidase (allopurinol) or nitric oxide synthase (L-NAME) with and without Sar[D-Phe^8^]des-Arg^9^-BK (20 µM; B_1_R agonist). Data show that the greater aortic O_2_^•−^ production induced by the B_1_R agonist was blocked only by apocynin.

**Conclusions:**

Activation of kinin B_1_R increased O_2_^•−^ through the activation of NADPH oxidase in the vasculature. Prolonged blockade of B_1_R restored cardiovascular, sensory and metabolic abnormalities by reducing oxidative stress and B_1_R gene expression in this model.

## Introduction

Recent evidence suggests a link between insulin resistance, oxidative stress, pain polyneuropathy and the overexpression of kinin B_1_ receptor [1], [2], [3]. Kinins are vasoactive peptides and pro-inflammatory pain mediators which act through the activation of two G-protein-coupled receptors (R), named B_1_ and B_2_. While the B_1_R has a low level of expression in healthy subjects, it is induced and overexpressed after exposure to pro-inflammatory cytokines, bacterial endotoxins and hyperglycaemia-induced oxidative stress [4], [5]. Bradykinin (BK) and Lys-BK are the natural agonists for the constitutive B_2_R, while the kininase I metabolites des-Arg^9^-BK and Lys-des-Arg^9^-BK are the selective agonists for the B_1_R [6].

Autoradiographic and molecular studies showed an increased density of B_1_R binding sites and mRNA in the brain, spinal cord and peripheral tissues of rats treated with D-Glucose (10% in drinking water) for a period of 4 and 12 weeks [2], [3], [7]. Glucose-fed rats displayed higher plasma levels of glucose and insulin, insulin resistance, arterial hypertension, enhanced production of superoxide anion (O_2_^•−^) in the heart and aorta [8], [9], [10] and pain polyneuropathy as assessed by the presence of tactile and cold allodynia [1], [2], [3]. Recently, we reported that all these abnormalities including B_1_R overexpression were reduced with a diet containing alpha-lipoic acid or N-Acetyl-L-Cysteine, two potent antioxidants [2], [3], supporting a link between the upregulation of B_1_R, diabetic complications and the oxidative stress. An acute treatment with B_1_R antagonists (LF22-0542, SSR240612 and R-715) reversed tactile and cold allodynia in high glucose feeding [1], [2]. However, only the brain penetrant B_1_R antagonist (LF22-0542) and not the peripherally acting R-715 decreased high systolic blood pressure in glucose-fed rats [2].

The present study was undertaken to determine the beneficial effect of a prolonged treatment (1 week) with the centrally and peripherally acting B_1_R antagonist SSR240612 on the main features and complications of diabetes in high glucose feeding. It is hypothesised that activation of B_1_R increases oxidative stress (aortic O_2_^•−^) and that its prolonged inhibition reverses oxidative stress and the subsequent upregulation of B_1_R which is responsible for arterial hypertension and pain polyneuropathy. The source of O_2_^•−^ was identified with the use of specific inhibitors of oxidative enzymes. The status of the antioxidant defence was determined by measuring the vascular expression of two selected antioxidant enzymes, superoxide dismutase (MnSOD) and catalase. MnSOD metabolises O_2_^•−^ to hydrogen peroxide which is converted to water by catalase. The data highlight a detrimental role for B_1_R in diabetes through a mechanism involving the oxidative stress and NADPH oxidase.

## Materials and Methods

### Animals and Procedures

Young male Sprague-Dawley rats (24–28 days old weighting 50–75 g, Charles River Laboratories, St-Constant, Quebec, Canada) were housed two per cage, under controlled conditions of temperature (22°C) and humidity (43%), on a 12-hour light-dark cycle and allowed free access to normal chow diet and tap water (control rats) or 10% D-glucose in the drinking water during 8 or 12 weeks for chronic and acute studies, respectively All research procedures and the care of the animals were in compliance with the guiding principles for animal experimentation as enunciated by the Canadian Council on Animal Care and were approved by the Animal Care Committee of our University (CDEA approval ID: 09-066).

### Acute effect of SSR240612 on blood pressure

A first series of experiments was performed in 12-week glucose-fed rats to assess the acute effects of several doses of SSR240612 on systolic blood pressure in order to select the optimal dose for chronic experiment. SSR240612 was administered by gavage at doses of 3, 10 and 30 mg/kg and effects were measured up to 48 h post-administration in unanaesthetized rats. At the end of this protocol, rats were euthanized by CO_2_ inhalation. Doses were selected on the basis of previous studies performed in various in vivo models of inflammation, pain and diabetes in rats and mice [11], [12], [13]. Moreover, these doses of SSR240612 were found appropriate to block acutely allodynia in the model of glucose-fed rats [1].

### Chronic effect of SSR240612 on blood pressure, allodynia and other parameters

These studies were carried out in 4 groups of 8-week glucose feeding and control rats (diabetic and control ± vehicle, diabetic and control ± SSR240612). The dose of 10 mg/kg SSR240612 was selected for the chronic study on the basis of the dose-response curve constructed on blood pressure (present study) and allodynia [1]. This dose was administered by gavage once a day in the morning for one week in control rats and in rats fed with D-Glucose. Thus, effects of 10 mg/kg of SSR240612 were determined on allodynia and arterial hypertension at 0 h, 3 h, 6 h, 24 h, 48 h, 72 h, 96 h, 120 h, 144 h and 168 h post-gavage. On day 7, overnight-fasted rats were anaesthetized and then euthanized by CO_2_ inhalation, 3 h after the last treatment with SSR240612, to collect tissues and blood samples for biochemical and molecular studies.

### Measurement of plasma glucose, insulin and insulin resistance

At the end of protocol, overnight-fasted rats were slightly anaesthetized with CO_2_ inhalation and blood was rapidly collected from sectioned carotid arteries and immediately transferred into a chilled tube of 6 ml containing 10.8 mg EDTA. The plasma was obtained by centrifugation and kept frozen at −20°C for the later measurement of glucose with a glucometer Accu-Chek (Roche Diagnostics Inc, Laval, Quebec, Canada) and insulin by radioimmunoassay (rat insulin RIA kit, Linco Research, St Charles, MO, USA) using 100 µl of plasma. The Homeostasis Model Assessment index (HOMA) was used as an index of insulin resistance and calculated with the following formula: [insulin (µU/ml) x glucose (mM)/22.5] [14].

### Measurement of systolic blood pressure

Systolic arterial blood pressure was measured by tail-cuff plethysmography (Harvard Apparatus Ltd, Kent, UK) with the use of a cuff placed around the tail and registered on a MacLab/8 system. For each measurement, three individual readings were averaged [2], [3].

### Measurement of Allodynia

Tactile and cold allodynia were assessed with the rats placed on a wire mesh floor beneath an inverted plastic cage. The rats were allowed to adapt for about 15 min or until explorative behaviour ceased. Tactile allodynia was assessed by measuring the hindpaw withdrawal threshold to the application of a calibrated series of six von Frey filaments (bending forces of 2, 4, 6, 8, 10 and 15 g) applied perpendicularly to the mid-plantar surface as described previously [1], [2], [3]. Cold allodynia was assessed using the acetone drop method applied to the plantar surface of the hindpaws as previously described [1], [2], [3]. The frequency of paw withdrawal was expressed as a percentage (the number of paw withdrawals ÷ number of trials ×100).

### Measurement of superoxide anion and NADPH oxidase activity in the aorta

Superoxide anion (O_2_^•−^) production was measured in frozen isolated thoracic aorta rings using the lucigenin-enhanced chemiluminescence method as described previously [15], [16]. Briefly, isolated aortas cut in 2–5 mm rings were pre-incubated at 37°C for 30 min in Krebs-HEPES buffer (saturated with 95% O_2_ and 5% CO_2_). Aortic rings were then transferred in duplicate to glass scintillation vials containing 200 µl of lucigenin (5 µM), which was previously dark adapted for 30 min. The chemiluminescence was recorded every minute for 10 min at room temperature using a liquid scintillation counter (Wallac 1409; Perkin Elmer Life Science, St Laurent, Quebec, Canada). Lucigenin counts were expressed as counts per minute per milligram of dry weight tissue (cpm/mg). The estimation of NADPH oxidase activity was achieved by adding to the aorta vials NADPH (10^−4^ M) before counting for another 6 min. Basal superoxide-induced luminescence was subtracted from the luminescence value induced by NADPH. Background counts were determined from vessel-free incubation media containing lucigenin and subtracted from the readings obtained with vessels.

Experiments designed to study the source of O_2_^•−^ were carried out as indicated above in freshly isolated aorta with the addition of vehicle or one of the following inhibitors: N^ω^-nitro-L-arginine methyl ester (L-NAME,100 µM, nitric oxide synthase inhibitor) [17], allopurinol (100 µM, xanthine oxidase inhibitor) [16], apocynin (10 µM, NADPH oxidase inhibitor) [18]. One hour later, the B_1_R agonist Sar[D-Phe^8^]des-Arg^9^-BK (20 µM) [19] was added to the solution for a further period of 15 min. In vivo experiments were also carried out in glucose-fed rats in which the B_1_R agonist Sar[D-Phe^8^]des-Arg^9^-BK (1 mg/kg, i.p.) was administered 30 min after apocynin (50 mg/kg, i.p.). After a systemic exposure of 30 min to the B_1_R agonist or its vehicle, rats were sacrificed under CO_2_ inhalation, the aortas isolated and processed for O_2_^•−^ measurement as indicated above.

The in situ level of O_2_^•−^ in the aorta was also evaluated by the oxidative fluorescent dye dihydroethidine as described earlier [20]. Cells are permeable to dihydroethidine and, in the presence of O_2_^•−^, it is oxidized to fluorescent ethidium bromide (EtBr) which is trapped by intercalation with DNA. EtBr is excited at 518 nm with an emission spectrum of 605 nm. Unfixed frozen aorta segments from the four experimental groups of rats (4 controls, 4 controls + SSR240612, 4 glucose-treated, 4 glucose-treated + SSR240612) were cut into 20-µm thick sections and placed on glass slides. Dihydroethidine (2 µM) was applied to tissue sections and coverslipped. The slides were then incubated in a light-protected humidified chamber at 37°C for 30 min. Images were obtained with a Leica TCS SP confocal microscope equipped with an argon laser (Leica microsystem Co., Germany). Tissues from the four groups were processed and imaged in parallel. Laser settings were identical for acquisition of images from all sections. Computer based analysis was performed with Image J software and calculated by the following equation: I = ∑ I/(A/N), where I is the fluorescence intensity, ∑ I is the summation of all nuclei intensity, A is the total area of the nuclei, and N is the number of nuclei used. Data are expressed as an average of total nuclei fluorescence quantified in triplicate of 4 rats.

### Real-time quantitative polymerase chain reaction (qRT-PCR)

Once the blood was collected after sacrifice, approximately 10 mg of each isolated tissue (thoracic aorta and spinal cord, liver, gastrocnemius muscle) were put in RNAlater stabilisation reagent (QIAGEN, Valencia, CA, USA). Total RNAs were extracted from tissue according to the manufacturer's instructions. First-strand cDNA synthesized from 400 ng total RNA with random hexamer primers was used as template for each reaction with the QuantiTect Rev Transcription Kit (QIAGEN). qRT-PCR was performed in SYBR Green Master mix (QIAGEN) with 300 nM of each primer and signal detected using a Mx3000p device (Stratagene, La Jolla, CA, USA) as described [21]. For standardization and quantification, rat 18S was amplified simultaneously. The primer pairs were designed by Vector NTI software (Table 1).

**Table 1:** Primer pairs used in qRT-PCR analysis.

Gene	Sequences	Position	Gen Bank
B_1_ receptor forward	5′GCA GCG CTT AAC CAT AGC GGA AAT 3′	367–391	NM_030851
B_1_ receptor reverse	5′CCA GTT GAA ACG GTT CCC GAT GTT 3′	478–454	NM_030851
18S forward	5′TCA ACT TTC GAT GGT AGT CGC CGT 3′	363–386	X01117
18S reverse	5′TCC TTG GAT GTG GTA GCC GTT TCT 3′	470–447	X01117
MnSOD forward	5′ACG CGA CCT ACG TGA ACA ATC TGA 3′	175–198	Y00497
MnSOD reverse	5′TCC AGC AAC TCT CCT TTG GGT TCT 3′	367–344	Y00497

PCR conditions were: 95°C for 15 min, followed by 46 cycles at 94°C for 15 s, 60°C for 30 s and 72°C for 30 s. The cycle threshold (Ct) value represents the cycle number at which a fluorescent signal rises statistically above background [22]. The relative quantification of gene expression was analyzed by the 2^−ΔΔCt^ method [23].

### Density and distribution of kinin B_1_ receptors by autoradiography

After sacrifice of rats, part of the thoracic spinal cord (T3–T7) was immediately frozen in 2-methylbutane cooled at −45 to −55°C with liquid nitrogen and kept at −80°C. Spinal cords were mounted in a gelatine block, serially cut into 20 µm thick coronal sections on a cryostat and stored at −80°C for 1 month. Thereafter sections were thawed at room temperature, pre-incubated for 10 min in 25 mM PIPES-NH_4_OH buffer (pH 7.4) and then incubated at room temperature for 90 min in the same buffer containing peptidase inhibitors and 200 pM of [^125^I]-HPP-desArg^10^-Hoe 140 [2], [3], [24]. The non-specific binding was determined in the presence of 1 µM of the B_1_ receptor antagonist: R715 (AcLys[D-ßNal^7^,Ile^8^]des-Arg^9^-BK [6]. Kodak Scientific Imaging Films BIOMAX™ MR® (Amersham Pharmacia Biotech Canada) were juxtaposed onto the slides in the presence of [^125^I]-microscales and exposed at room temperature for 5–7 days. Autoradiograms were quantified by densitometry using an MCID™ image analysis system. A standard curve from [^125^I]-microscales was used to convert density levels into fentomoles per milligram of protein [25]. Specific binding was determined by subtracting values of non-specific binding from that of total binding. Total binding and non-specific binding were measured separately on 4 sections per rat with 6–8 rats per group.

### Western blot analysis

Western blot analysis of protein expression was performed as described earlier [26]. After sodium dodecyl sulfate polyacrylamide gel electrophoresis (SDS-PAGE), the separated proteins were electrophoretically wet transferred to a nitrocellulose membrane (Bio-Rad) at 100 V for 1 h. After transfer, the membranes were washed twice in PBS-Tween 20 and incubated in PBS containing 5% skim milk at room temperature for 1 h. The blots were cut in pieces according to the molecular weight of the protein and then incubated with the specific antibodies for MnSOD (1/500, cat number: sc-18503), catalase (1/500, cat number: sc-34285), dynein (1/5000, cat number: sc-13524) and β-actin (1/10000, cat number A5441) in PBS-Tween 20 at 4°C overnight. Dynein and β-actin were used as standard proteins. After three washings in PBS-Tween 20 buffer, the membranes were incubated for 1 h at room temperature in PBS-Tween 20 containing 5% milk with secondary antibody that is bovine anti-goat IgG-HRP (1/5000, cat number: sc-2350) or anti-mouse IgG-HRP (1/5000, cat number sc-2005) (for β-actin and dynein). β-actin antibody was purchased from Sigma-Aldrich Canada while other antibodies were from Santa Cruz Biotechnology, CA, USA. The blots were then washed three times with PBS-Tween 20 before the reaction with enhanced-chemiluminescence, Western blotting detection reagents (Amersham). A quantitative analysis of the protein was performed by densitometric scanning of the autoradiographs employing the enhanced laser densitometer, LKB Ultroscan XL, and quantified using the gel-scan XL evaluation software (version 2.1) from Pharmacia (Baie d'Urfé, Quebec, Canada).

### Drugs

The selective non-peptide B_1_R antagonist SSR240612 ((2R)-2-[((3R)-3-(1,3-benzodioxol-5-yl)-3-{[(6-methoxy-2-naphtyl)sulfonyl]amino}propanoyl)amino]-3-(4-{[(2R,6S)-2,6-dimethylpiperidinyl]methyl}phenyl)-N-isopropyl-N-methylpropanamide,fumarate) wasobtained from Sanofi-Aventis R&D (Montpellier, France) [12]. Sar[D-Phe^8^]des-Arg^9^-BK and HPP-des-Arg^10^-Hoe140 (3-(4hydroxyphenyl)propionyl-desArg^9^-D-Arg^0^[Hyp^3^,Thi^5^,D-Tic^7^,Oic^8^]Bradykinin) were synthesized at the Research Institute of Biotechnology, National Research Council of Canada (Montreal, Quebec, Canada). R-715 was kindly provided by Dr D. Regoli (Pharmacology, University of Ferrara, Italy). Iodination of HPP-des-Arg^10^-Hoe140 was performed with the chloramine T method as described earlier [24]. SSR240612 was dissolved in dimethyl sulfoxide (DMSO, 0.5% v/v) and then ethanol (5% v/v) and Tween-80 (5% v/v) were added in this sequence. The solution was completed in distilled water. The drug was administered orally by gavage in a volume of 1 ml per 100 g of body weight. D-Glucose, apocynin, allopurinol, NADPH, lucigenin and L-NAME were purchased from Sigma-Aldrich Canada. Dihydroethidine was obtained from Molecular Probes (Invitrogen Corporation, Carisbad, CA, USA) and suspended in DMSO at a concentration of 10^−3^ M, and stored at −20°C until use. Subsequent dilutions were made in PBS. All other chemicals used were purchased from standard commercial suppliers and were of analytical grade.

### Statistical analysis of data

Data are expressed as mean ± s.e.m of values obtained from (n) rats in each group. Statistical analysis of data was calculated with GraphPad Prism (version 4.00) software. Statistical differences were evaluated with Student's t-test on unpaired samples (B_1_R binding sites). Multiple comparisons were analysed using one-way or two-way analysis of variance (ANOVA), followed by the Bonferroni post-test. Only probability values (P) less than 0.05 were considered to be statistically significant.

## Results

### Acute effect of SSR240612 on systolic blood pressure

**Figure 1:**
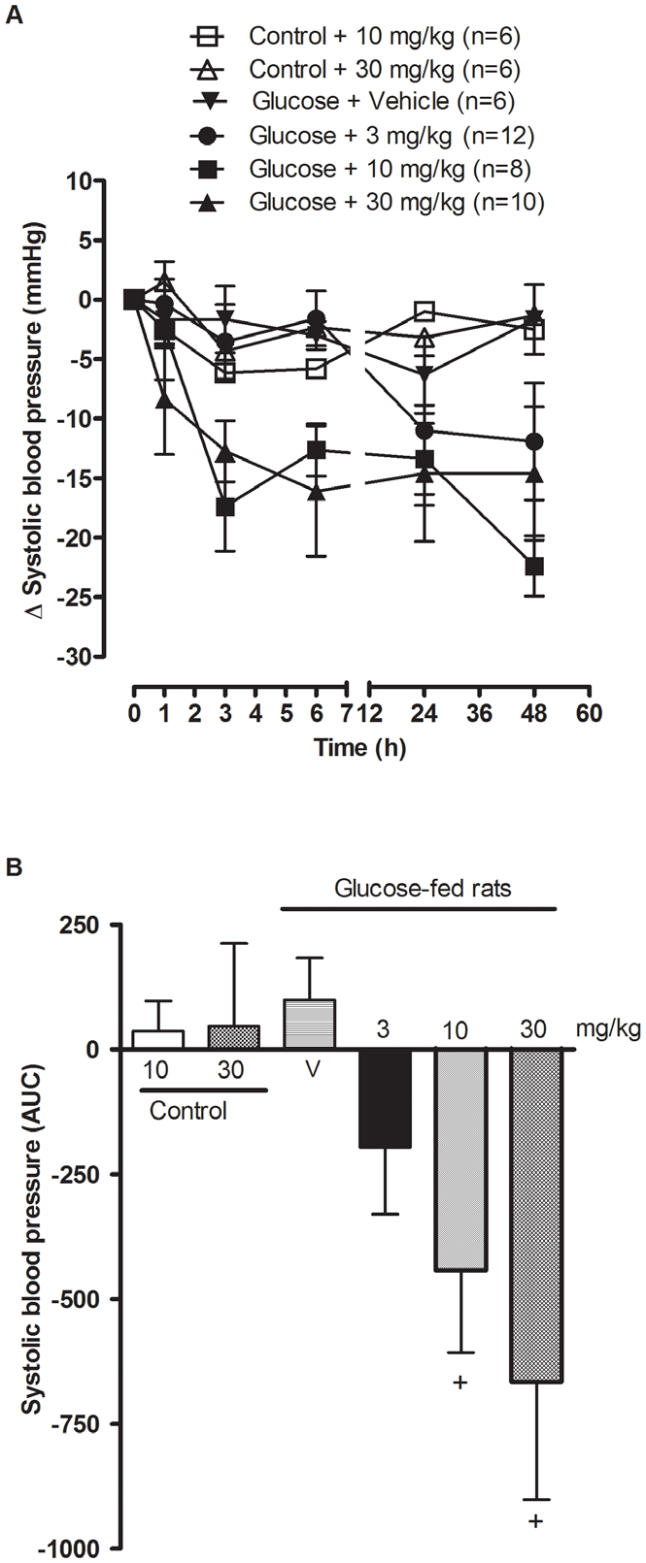
Acute effect of orally administered SSR240612 on systolic blood pressure in 12-week glucose-fed rats. Doses of 10 mg/kg and 30 mg/kg of SSR240612 were given to control rats and doses of 3, 10, 30 mg/kg of SSR240612 or vehicle were given to glucose-fed rats. Data are mean ± s.e.m of values obtained from (n) rats and represent changes of systolic blood pressure in mmHg (A) or Area Under the Curve (B). Statistical comparison with vehicle (V) in glucose-fed rats (+) is indicated by + P< 0.05.

Systolic blood pressure was significantly increased (P<0.001, n = 6–12) in the four groups of 12-week glucose-treated rats when compared to control rats and was dose-dependently reduced by the oral administration of SSR240612 (Baseline values of Glucose + vehicle: 151.2±5.7 mmHg; Glucose + SSR240612 (3 mg/kg): 152.2±2.0 mmHg; Glucose + SSR240612 (10 mg/kg): 160.4±3.2 mmHg; Glucose + SSR240612 (30 mg/kg): 156.5±4.7 mmHg; Control + SSR240612 (10 mg/kg): 125.3±3.3 mmHg; Control + SSR240612 (30 mg/kg): 129.7±1.9 mmHg) (Figure 1A). When the area under the curve was measured between 0 h to 48 h post-administration, the dose of 3 mg/kg did not reach statistical significance. However, doses of 10 and 30 mg/kg SSR240612 decreased significantly high blood pressure when compared with vehicle (P<0.05). In contrast, doses of 10 and 30 mg/kg SSR240612 administered to control rats had no significant effect on systolic blood pressure (Figure 1B). Therefore, the dose of 10 mg/kg SSR240612 was selected for chronic treatment in the remainder of the study.

### Chronic effect of SSR240612 on systolic blood pressure

**Figure 2:**
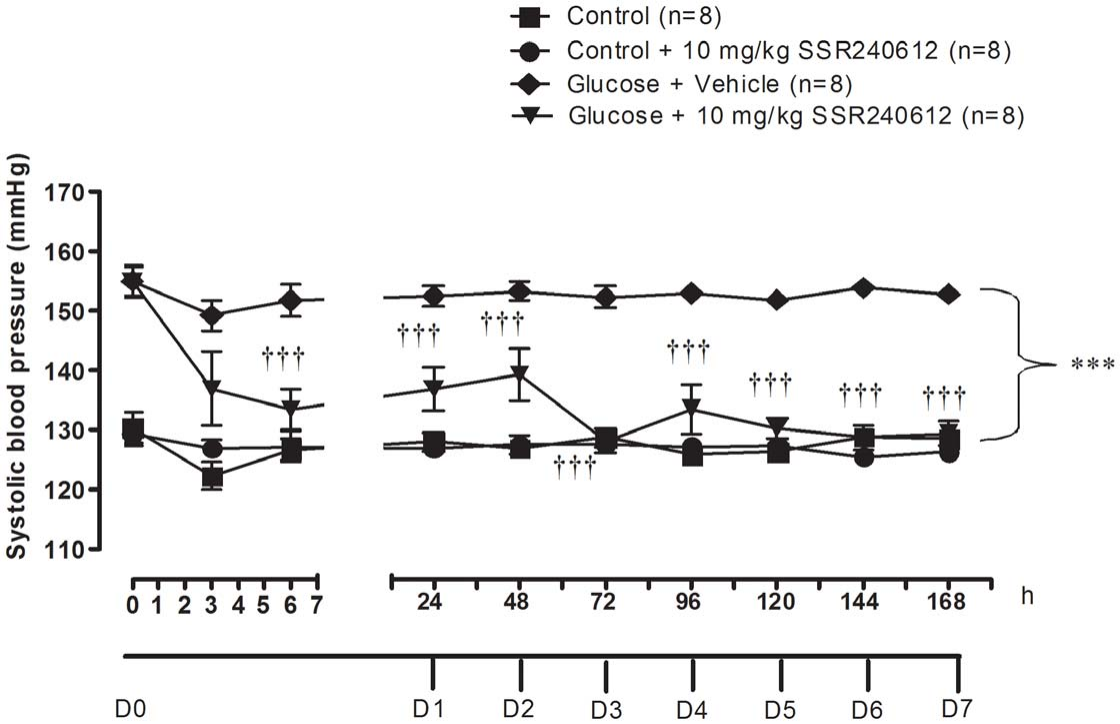
Effect of orally administered SSR240612 (10 mg/kg/day for 7 days) on systolic blood pressure in glucose-fed rats. From day (D) 1 to 7, measurements were taken prior to the morning treatment with SSR240612. Data are mean ± s.e.m of values obtained from (n) rats. Statistical comparison with controls (*) or glucose-fed rats + vehicle (†) is indicated by ***†††P< 0.001.

As shown in figure 2, systolic blood pressure was significantly higher (P<0.001) in glucose-fed rats when compared to age-matched control rats. A one-week treatment with SSR240612 (10 mg/kg) reduced significantly high blood pressure in glucose-fed rats at 6 h on day 0 and during the remaining period of treatment when compared to age-matched glucose-fed rats receiving the vehicle. The reduction of blood pressure was incomplete during the premier 2 days of treatment but became sustained and reached control values from day 3 to day 7. In contrast, the same treatment with SSR240612 for a period of one week had no significant effect on systolic blood pressure in control rats when compared to untreated control rats.

### Chronic effect of SSR240612 on allodynia

**Figure 3:**
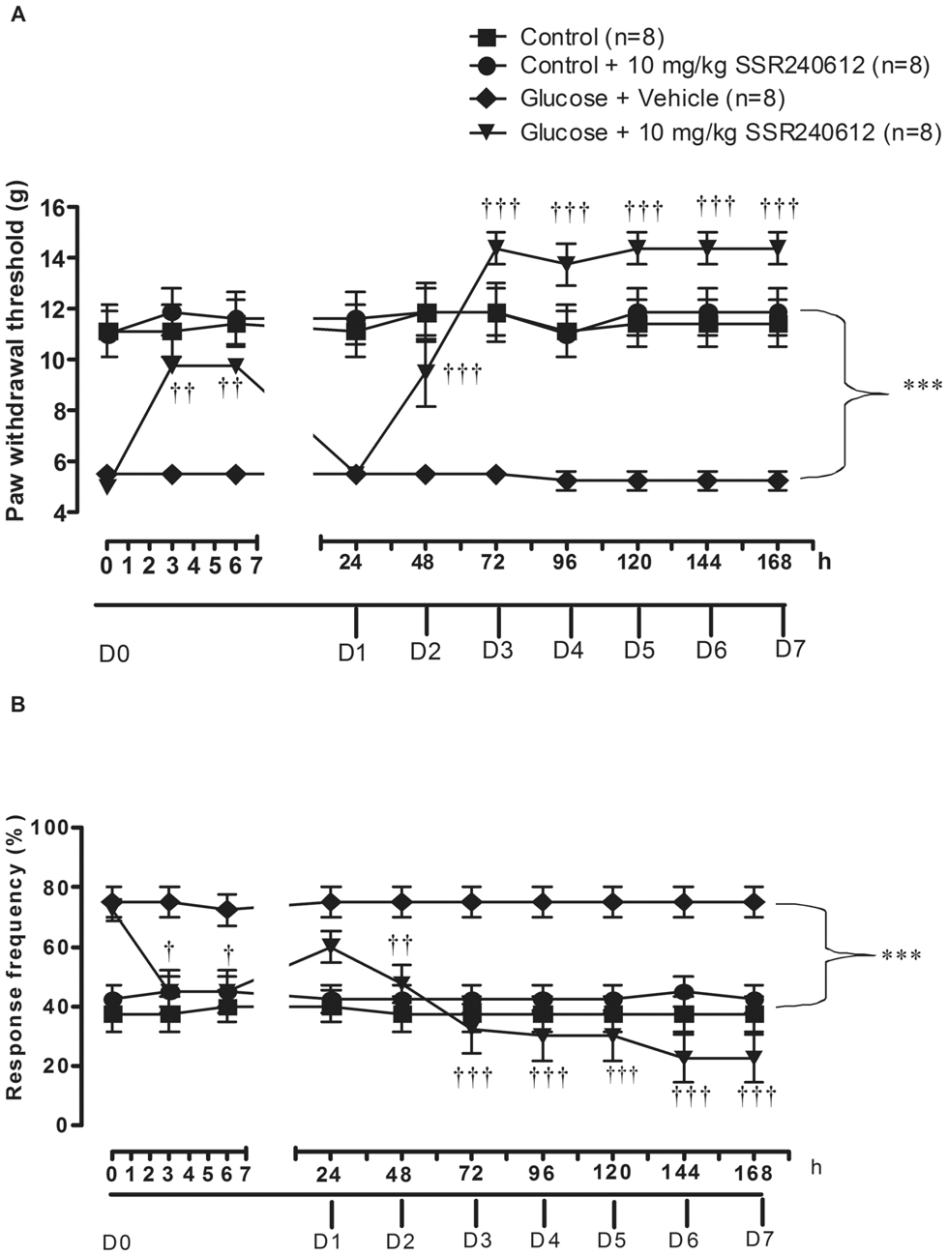
Effect of orally administered SSR240612 (10 mg/kg/day for 7 days) on (A) tactile allodynia and (B) cold allodynia in glucose-fed rats. From day (D) 1 to 7, measurements were taken prior to the morning treatment with SSR240612. Data are mean ± s.e.m of values obtained from (n) rats. Statistical comparison with controls (*) or glucose-fed rats + vehicle (†) is indicated by †P< 0.05, ††P< 0.01, ***†††P< 0.001.

As shown in figure 3A, glucose-fed rats displayed significant and sustained tactile allodynia (P<0.001) when compared to age-matched control rats from day 0 to day 7. Chronic treatment with 10 mg/kg SSR240612 caused a significant reduction of tactile allodynia in glucose-fed rats at 3 and 6 h post-gavage on day 0 when compared to glucose-fed rats treated with vehicle. The inhibition was entirely reversible at 24 h but not on the subsequent days until the completion of the treatment on day 7. The higher paw withdrawal threshold occurring between day 3 to day 7 in glucose-fed rats treated with SSR240612 was not significantly different from control values. Finally, daily administration of 10 mg/kg SSR240612 for 7 days had no significant effect on paw-withdrawal threshold to tactile stimulation in control rats when compared to untreated control rats.

As shown in figure 3B, glucose-fed rats also displayed significant cold allodynia (P<0.001) when compared to age-matched control rats from day 0 to day 7. Significant reduction of cold allodynia was seen at 3 and 6 h after treatment with 10 mg/kg SSR240612 on day 0 when compared to glucose-fed rats treated with vehicle. This inhibition was no longer significant at 24 h. On the following days, daily treatment with SSR240612 led to a striking and irreversible inhibition of cold allodynia. From day 2 to day 7, response frequency to cold stimulation was not significantly different between control and glucose-fed rats treated with SSR240612. In contrast, the same treatment with SSR240612 for one week had no significant effect on paw-withdrawal threshold to cold stimulation in control rats when compared to untreated control rats.

### Chronic effect of SSR240612 on various parameters

As shown in table 2, body weight was not significantly different between the four groups. Plasma levels of glucose and insulin were significantly increased in rats fed with 10% D-Glucose when compared with age-matched control rats. Plasma glucose levels in glucose-fed rats treated for 1 week with 10 mg/kg SSR240612 were not significantly different from control values. High plasma insulin levels were significantly reduced in glucose-fed rats treated with SSR240612 when compared with glucose-fed rats treated with vehicle. Insulin resistance as assessed by the HOMA index was increased by 5.6-fold in glucose-fed rats when compared with age-matched control rats. This value was significantly reduced though not completely normalised by one-week treatment with SSR240612. The same regimen with SSR240612 failed to affect plasma levels of glucose and insulin and the HOMA index in control rats. Water intake was increased by 2-fold in glucose-fed rats and this was compensated by a 33% reduction of food intake. SSR240612 treatment for 7 days had no effect on drinking or eating behaviour in both glucose-fed rats and controls rats (Table 2).

**Table 2:** Effects of SSR240612 (10 mg/kg/day ×7 days) in rats treated with glucose.

Parameters	Controln = 6	Control + SSR240612 n = 6	Glucose-fed + Vehiclen = 8	Glucose-fed + SSR240612n = 8
Body weight (g)	392.0±12.3	397.2±5.9	408.4±7.3	387.6±12.9
Plasma glucose (mmol/L)	5.8±0.2	6.3±0.2	7.8±0.8 ^*^	6.6±0.4
Plasma insulin (ng/ml)	1.0±0.3	0.8±0.2	7.0±1.4 ^***^	2.1±0.3 ^** †††^
HOMA index	5.2±1.3	4.9±1.1	29.2±2.8 ^***^	10.4±1.8 ^* †††^
Drinking (ml)	58.2±2.2	59.7±1.2	119.1±5.0 ^**^	112.6±8.5 ^**^
Food intake (g)	30.9±0.2	30.6±0.2	20.7±1.1 ^**^	22.5±0.8 ^**^

Values represent the mean ± s.e.m of (n) rats. Statistical comparison to control rats (*) or to glucose + vehicle (^†^) is indicated by ^*^ P<0.05;^**^ P<0.01; ^*** †††^ P<0.001.

### Chronic effect of SSR240612 on B_1_R binding sites and B_1_R mRNA in spinal cord

**Figure 4:**
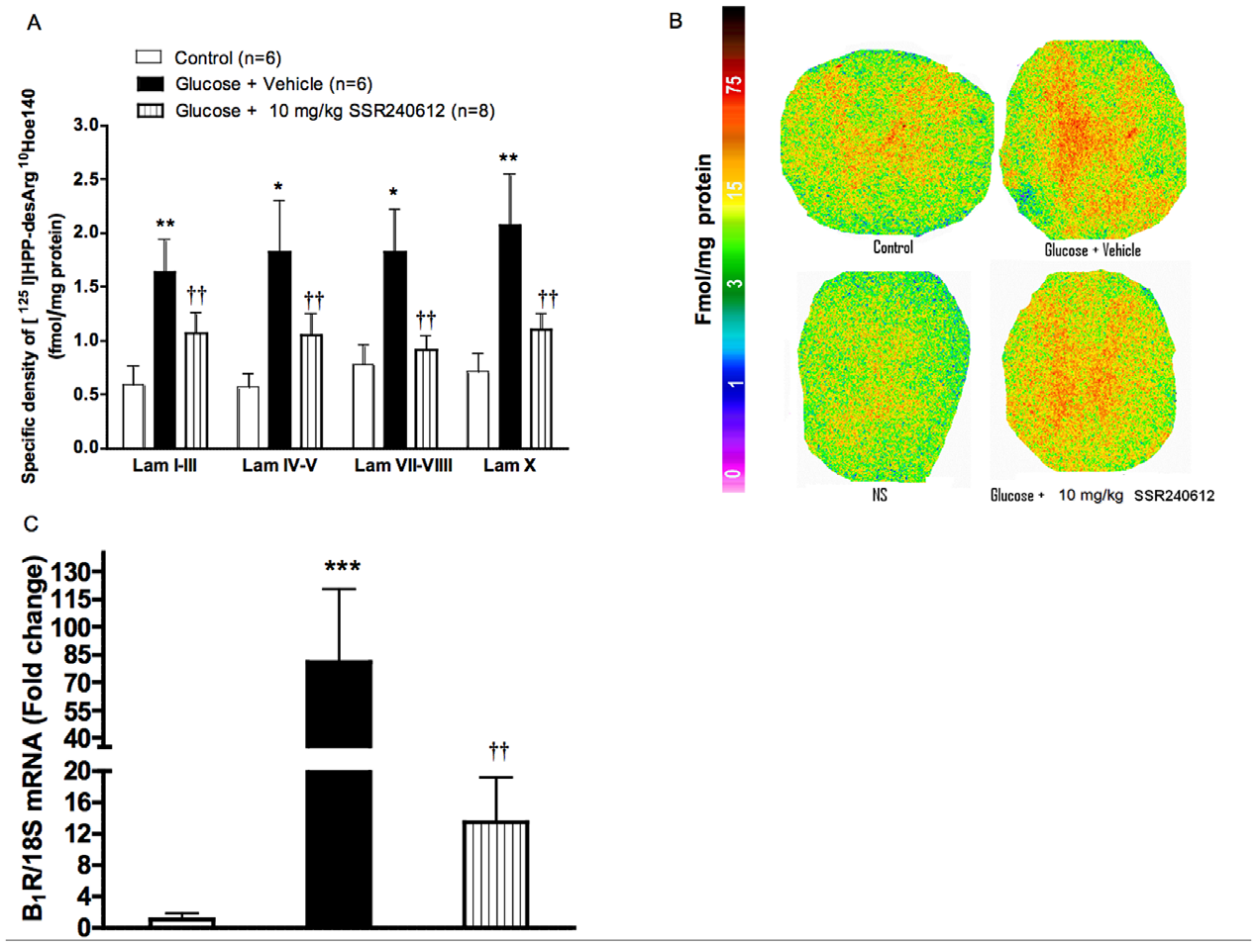
Effect of orally administered SSR240612 (10 mg/kg/day for 7 days) on B_1_R expression in the thoracic spinal cord. A: Quantification of specific density of [^125^I]-HPP-desArg^10^-Hoe 140 to B_1_R in various spinal cord laminae (Lam). B: Autoradiograms of thoracic spinal cord for B_1_R. The non-specific binding (NS) was obtained by the co-addition of 1 µM R-715 (B_1_R antagonist) with [^125^I]-HPP-desArg^10^-Hoe 140. C: Bars represent fold change in gene expression for B_1_R. Data are mean ± s.e.m of values obtained from (n) rats. Statistical comparison with controls (*) or glucose-fed rats + vehicle (†) is indicated by * P< 0.05, **††P< 0.01, ***P< 0.001.

Quantitative autoradiography showed a significant increase of specific density of kinin B1R binding sites through laminae I to X of the thoracic spinal cord in glucose-fed rats when compared to age-matched control spinal cord (Figure 4A and 4B). This overexpression of B1R binding sites in glucose-fed rats was significantly reduced in all laminae by the one-week treatment with 10 mg/kg SSR240612. As shown in figure 4C, B1R mRNA was underexpressed in the spinal cord of control rats. In glucose-fed rats, however, B1R mRNA was increased by 80-fold. Again, the overexpression of B1R mRNA in the spinal cord of glucose-fed rats was significantly and markedly decreased by SSR240612.

### Chronic effect of SSR240612 on B_1_R mRNA levels in peripheral organs

**Figure 5:**
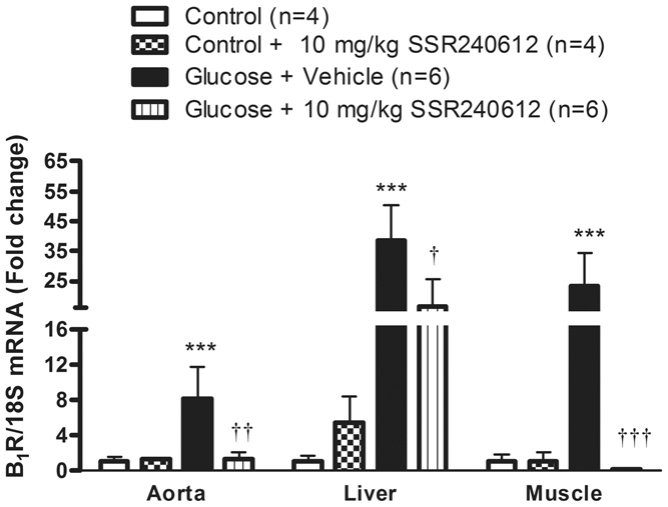
B_1_R mRNA levels in aorta, liver and skeletal muscle after orally administered SSR240612 (10 mg/kg/day for 7 days). Data are mean ± s.e.m of values obtained from (n) rats. Statistical comparison with controls (*) or glucose-fed rats + vehicle (†) is indicated by † P< 0.05, ††P< 0.01, ***†††P< 0.001.

Similarly to the spinal cord, levels of B1R mRNA were relatively low in aorta, liver and gastrocnemius muscle of control rats (Figure 5). However in glucose-fed rats, B1R mRNA levels were markedly and significantly upregulated in the same tissues. The one-week treatment with 10 mg/kg SSR240612 reversed completely B1R mRNA overexpression in aorta and skeletal muscle and reduced significantly B1R mRNA level in the liver of glucose-fed rats. The antagonist was without effect on the basal expression of B1R mRNA in control rats.

### Chronic effect of SSR240612 on oxidative stress

**Figure 6:**
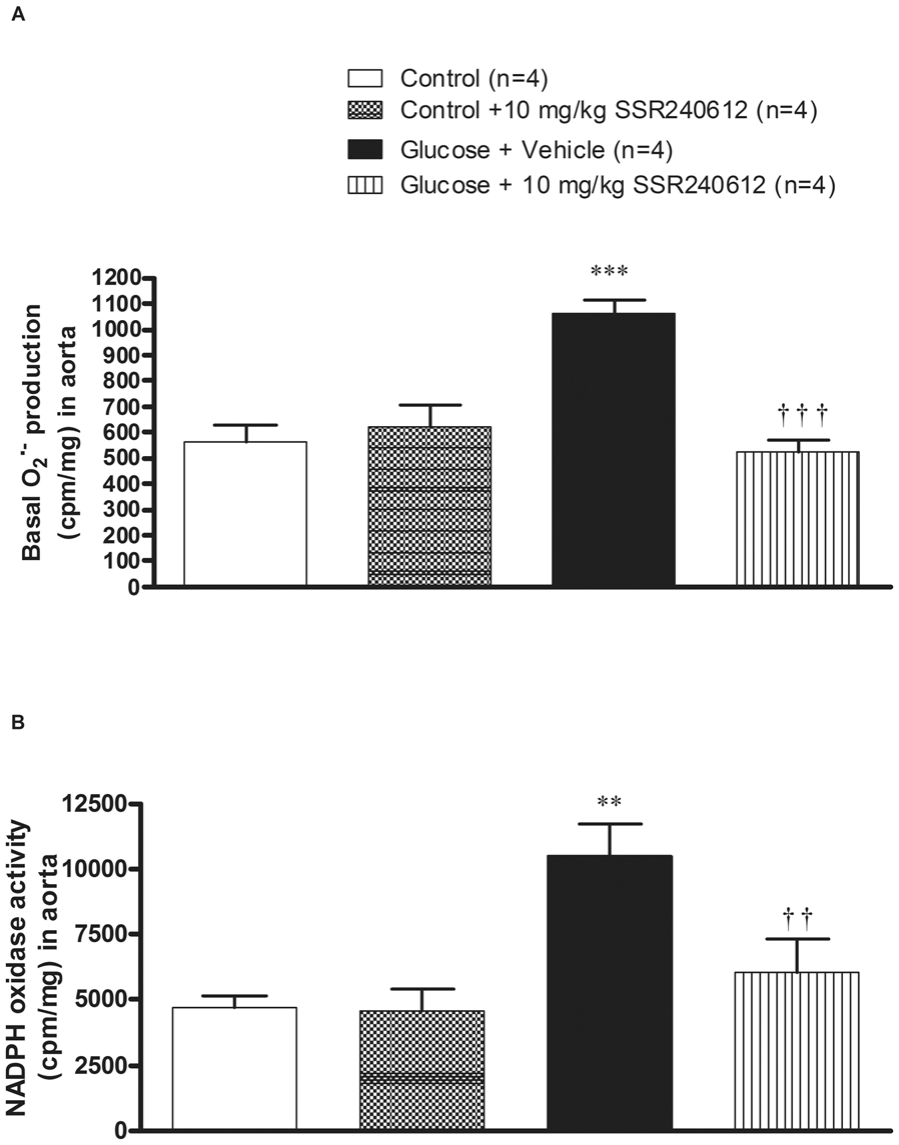
Effect of orally administered SSR240612 (10 mg/kg/day for 7 days) on oxidative stress. (A) Basal superoxide anion production, (B) NADPH oxidase activity in the aorta of glucose-fed rats. Data are mean ± s.e.m of values obtained from (n) rats. Statistical comparison with controls (*) or glucose-fed rats + vehicle (†) is indicated by **††P< 0.01, ***†††P< 0.001.

Effects of 8-week treatment with glucose on basal and NADPH-stimulated O_2_^•−^ production measured in the aorta using lucigenin-enhanced chemiluminescence are shown in figure 6. Glucose feeding resulted in a 1.9-fold increase of basal O_2_^•−^ production in the aorta when compared to control aorta (Figure 6A). The one-week treatment with SSR240612 normalised the higher production of O_2_^•−^ in glucose-fed rats to control levels. SSR240612 failed, however, to alter basal O_2_^•−^ production in the aorta of control rats (Figure 6A). Moreover, NADPH oxidase activity was significantly increased by 2-fold in the aorta of glucose-fed rats. Again, the increase of NADPH oxidase activity in glucose-fed rats was significantly reduced by the one-week treatment with SSR240612. The latter treatment with the B_1_R antagonist failed to alter O_2_^•−^ production induced by NADPH in the aorta of control rats (Figure 6B).

**Figure 7:**
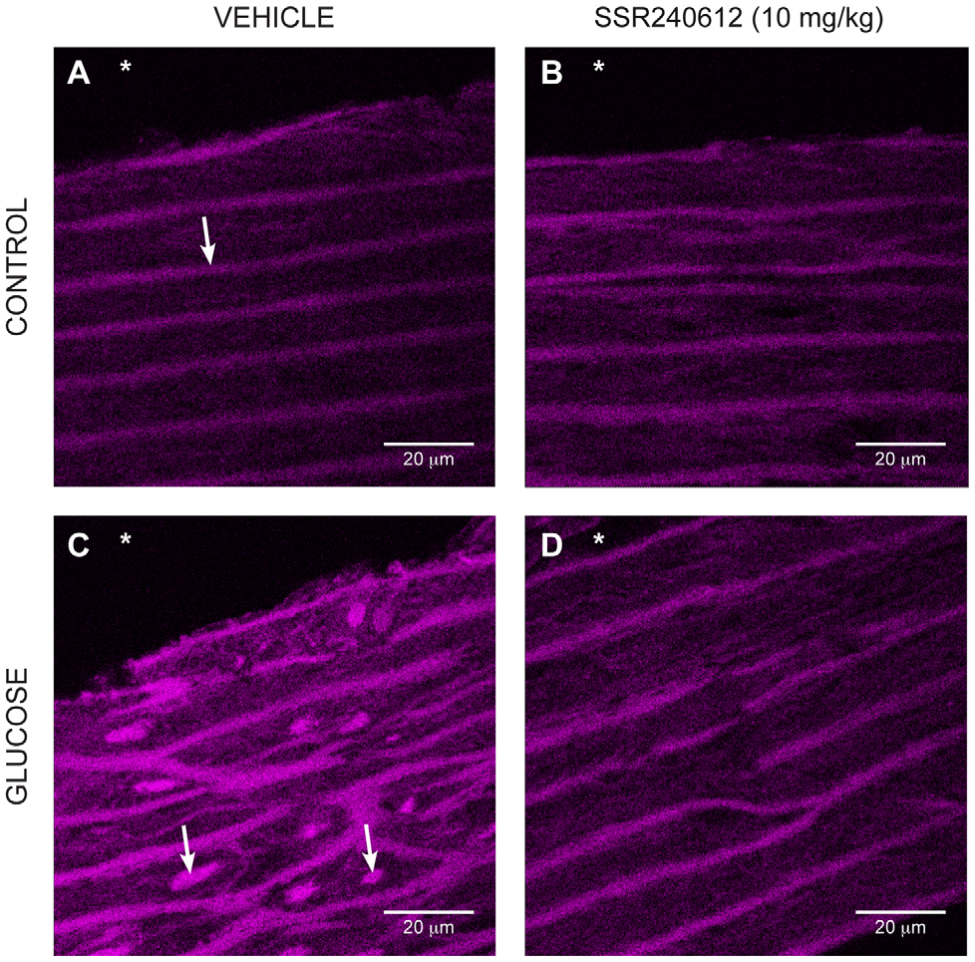
Superoxide anion production on histological sections of the aorta marked with dihydroethidine. Control (A, B) and glucose-treated rats (C, D) after 7-day treatment with Vehicle (A, C) or 10 mg/kg/day SSR240612 (B, D). Asterisk (*) indicates the lumen side of the section. The arrow (in A) represents the elastic lamina of the smooth muscle cells while the two arrows (in C) show the increase of fluorescent ethidium bromide in the nucleus of smooth muscle cells in the aorta of glucose-treated rat. The fluorescent marker is reduced after treatment with SSR240612 (in D). Scale bar is 20 µm in each panel. Images are representative of 4 aortas in each group.

**Figure 8:**
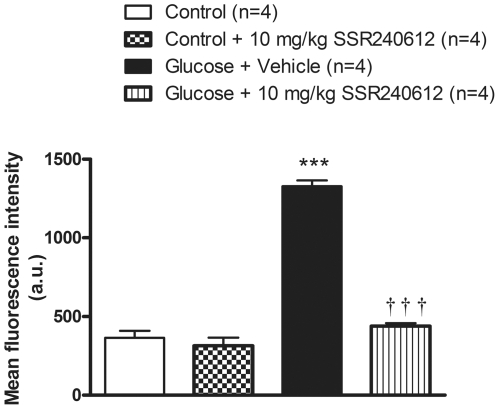
Fluorescence intensities of superoxide (dihydroethidium) staining in aortic sections from control and 8-week glucose-treated rats shown in **Figure 7**. Data are expressed as fluorescence intensity in arbitrary unit (a.u.) and represent the mean ± s.e.m of 4 rats in each group. Statistical comparison with controls (*) or glucose-fed rats + vehicle (†) is indicated by ***†††P< 0.001.

The production of O2^•−^ evaluated with the oxidative fluorescent dye dihydroethidine was also markedly increased in vascular smooth muscle cells of the aorta in glucose-fed rats (Figures 7C and 8). Daily treatment with 10 mg/kg SSR240612 for a week abolished the fluorescent labelling seen in the aorta of glucose-fed rats to control levels (Figures 7D and 8). However, the weak labelling displayed in the aorta of control rats was not affected by the B1R antagonist (Figures 7A-B and 8).

### Pro-oxidative effect of B_1_R

**Figure 9:**
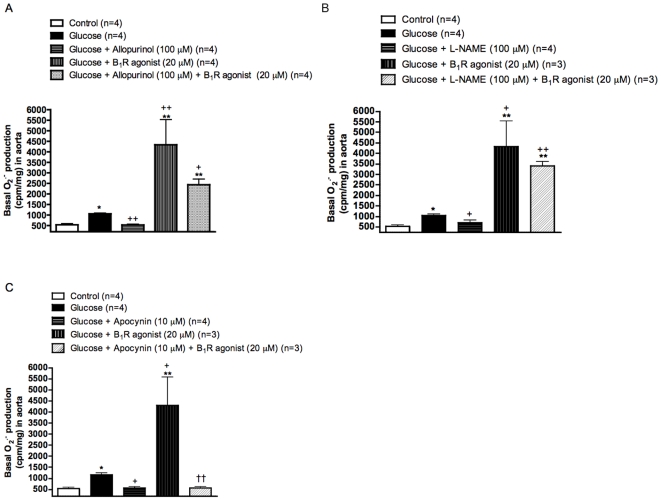
Effects of *in vitro* B_1_R activation on superoxide anion production in the aorta of glucose-fed rats pre-treated or not with oxidative enzyme inhibitors. Basal and stimulated production of superoxide anion in the presence of the B_1_R agonist Sar[D-Phe^8^]des-Arg^9^-BK (20 µM) were measured in the presence of (A) Allopurinol (xanthine oxidase inhibitor), (B) L-NAME (inhibitor of all NOS isoforms) and (C) Apocynin (NADPH oxidase inhibitor) at the indicated concentrations (see methods). Data are mean ± s.e.m of values obtained from (n) rats. Statistical comparison with control (*), glucose (+) or Sar[D-Phe^8^]des-Arg^9^-BK (†) is indicated by * ^+^ P< 0.05, ** ^++ ††^P< 0.01.

To further substantiate the contribution of B1R in the production of O2^•−^, aortas from glucose-fed rats were incubated in the presence of the B1R agonist Sar[D-Phe8]des-Arg9-BK (20 µM). Results presented in figure 9 show that the B1R agonist enhanced by 4-fold the production of O2^•−^. Whereas the pro-oxidative response to Sar[D-Phe8]des-Arg9-BK was not significantly affected by allopurinol (xanthine oxidase inhibitor) and L-NAME (a non-selective inhibitor of all NOS isoforms), it was completely blocked by apocynin (NADPH oxidase inhibitor). Baseline values of O2^•−^ production in glucose-treated aortas were either slightly reduced (L-NAME) or normalized (apocynin and allopurinol) by these inhibitors.

**Figure 10:**
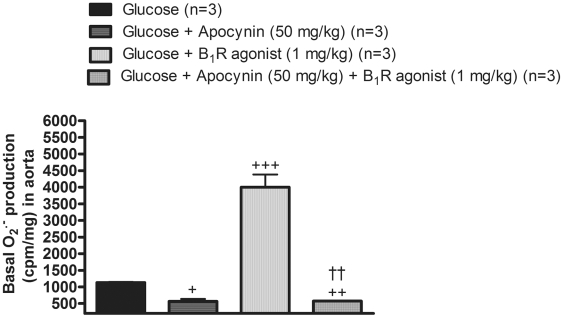
Effects of *in vivo* B_1_R activation on superoxide anion production in the aorta of glucose-fed rats pre-treated or not with the NADPH oxidase inhibitor. Basal and stimulated production of superoxide anion with the B_1_R agonist Sar[D-Phe^8^]des-Arg^9^-BK (1 mg/kg, i.p.). Apocynin (50 mg/kg, i.p.) was administered 30 min prior to the B_1_R agonist (see methods). Data are mean ± s.e.m of values obtained from (n) rats. Statistical comparison with glucose (+) or Sar[D-Phe^8^]des-Arg^9^-BK (†) is indicated by ^+^ P< 0.05, ^++ ††^P< 0.01, ^+++^P< 0.001.

The intraperitoneal administration of Sar[D-Phe8]des-Arg9-BK (1 mg/kg) in 8-week glucose-fed rats also enhanced by 4-fold the production of O2^•−^ in aorta (Figure 10). Similarly to the *in vitro* protocol, systemic treatment with apocynin (50 mg/kg) abolished the effect of the B1R agonist on the production of O2^•−^. It is worth mentioning that apocynin normalized the increasing effect of glucose on baseline O2^•−^ production. These findings suggest that B1R activation can increase the production of superoxide anion primarily through NADPH oxidase.

### Chronic effect of SSR240612 on superoxide dismutase and catalase expression

**Figure 11:**
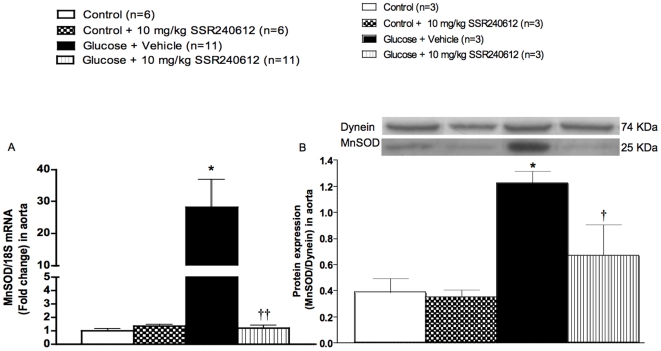
Effect of orally administered SSR240612 (10 mg/kg/day for 7 days) on vascular expression of superoxide dismutase. The expression of MnSOD was measured at (A) mRNA level by qRT-PCR, and (B) at protein level by Western blot in the aorta of glucose-fed rats. Data are mean ± s.e.m of values obtained from (n) rats. Statistical comparison with controls (*) or glucose-fed rats + vehicle (†) is indicated by *^†^P< 0.05, ^††^P< 0.01.

**Figure 12:**
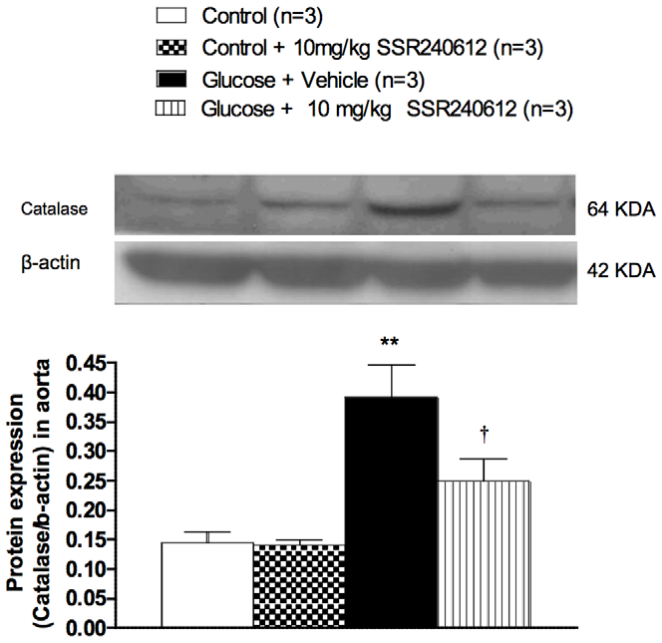
Effect of orally administered SSR240612 (10 mg/kg/day for 7 days) on vascular expression of catalase. The expression of catalase was measured at protein level by Western blot in the aorta of glucose-fed rats. Data are mean ± s.e.m of values obtained from (n) rats. Statistical comparison with controls (*) or glucose-fed rats + vehicle (†) is indicated by ^†^P< 0.05, ^**^P< 0.01.

The impact of SSR240612 was evaluated on the vascular antioxidant defence. Firstly, the mRNA and protein expressions of MnSOD were markedly increased in the aorta of glucose-fed rats when compared to age-matched control rats (Figure 11A and 11B). The up-regulation of this antioxidant enzyme was reversed (mRNA) or significantly reduced (protein) by the one-week treatment with 10 mg/kg SSR240612. Secondly, the protein expression of catalase was significantly increased in the aorta of 8-week glucose-fed rats and the one-week treatment with SSR240612 reduced it significantly (Figure 12). In contrast, the prolonged treatment with the B_1_R antagonist had no significant effect on MnSOD or catalase expression in the aorta of control rats (Figures 11–12).

## Discussion

This study provides the first demonstration that the activation of B_1_R increased the oxidative stress through the activation of NADPH oxidase in the vasculature and that the sustained inhibition of B_1_R for one-week with SSR240612 reversed the oxidative stress and the subsequent B_1_R upregulation in a model of insulin resistance. Indeed, activation of B_1_R with a selective agonist either *in vitro* or *in vivo* enhanced the production of aortic superoxide anion which was abolished by apocynin, a selective inhibitor of NADPH oxidase. The inhibition of B_1_R had positive outcome on diabetic complications in the model of glucose-fed rat. SSR240612 treatment had no effect on drinking and food intake in both control and glucose-fed rats, excluding an indirect effect of the drug on glucose intake.

Our findings on B_1_R-induced allodynia are in keeping with the role of B_1_R in hyperalgesia in inflammatory, diabetic and painful processes [27], [28], [29] and with the therapeutic effect of SSR240612 in neuropathic and inflammatory pain [12], [30].

### Relationship between oxidative stress, B_1_R and glucose-induced hypertension and allodynia

It is known that diets containing high refined carbohydrates such as fructose, sucrose and glucose elevate blood pressure in rats [10], [31], [32], [33]. Simple carbohydrate feeding to rat and high glucose infusion for 2 days elevate reactive oxygen species (ROS) [9], [34], [35]. Increased production of O_2_^•−^ is correlated with high blood pressure in glucose-fed rats [8], [9]. Alpha-lipoic acid attenuates aortic and heart mitochondrial O_2_^•−^ production in glucose-fed rats [7], [10] and reverses hypertension in fructose and glucose-fed rats [2], [10], [36]. The antioxidant N-acetyl-L-cysteine also prevents high blood pressure in fructose and glucose feeding rats [3], [37].

It is also well established that increased oxidative stress causes diabetic neuropathy, especially through the polyol pathway (high activity of the aldose reductase). Hyperglycemia induces nerve loss and reduces nerve velocity through oxidative stress. These problems are alleviated by alpha-lipoic acid [38]. In our model of high glucose feeding, alpha-lipoic acid and N-acetyl-L-cysteine were able to reduce simultaneously vascular oxidative stress, B_1_R upregulation, hypertension and allodynia [2], [3], [7]. This is consistent with the increased B_1_R mRNA and protein expression following a 12 h exposure of mesenteric vascular endothelial cells with 25 mM glucose [39]. The corollary of these findings is that the oxidative stress is likely the primary mechanism involved in the induction of B_1_R in the model of insulin resistance induced by high glucose intake. The oxidative stress can activate the nuclear factor kappa B (NF-κB) pathway [40], [41] which is directly involved in the upregulation of B_1_R [4]. B_1_R may therefore represent a molecular marker of the oxidative stress.

### Source of superoxide anion and pro-oxidative effect of B_1_R in glucose-fed rats

The NADPH oxidase is a predominant source of ROS production (O_2_^•−^) in cardiovascular tissues in response to high glucose, growth factors and vasoactive peptides [42], [43], [44]. NADPH oxidase activity and O_2_^•−^ levels were increased in cultured vascular smooth muscle cells exposed to high glucose concentration [45], in animal and clinical models of hypertension and diabetes [42], [44], [46]. In our study, the complete inhibition of increased glucose-induced superoxide anion by apocynin confirmed the predominant contribution of NADPH oxidase. However, the complete inhibition O_2_^•−^ levels with allopurinol and its partial inhibition with L-NAME suggest multiple sources of ROS in this model, including xanthine oxidase and uncoupling eNOS. Xanthine oxidase was also proposed as a source of ROS in the vasculature in models of hypertension [42]. Furthermore, the increased NADPH activity observed in the present study is in agreement with data reported in the aorta of db/db mice, a type 2 model of diabetes [46].

A key finding of the present study was the demonstration that a one-week treatment with SSR240612 reversed the vascular oxidative stress and normalized B_1_R up-regulation in glucose-fed rats. This contrasts with the acute treatment with SSR240612 which did not affect O_2_^•−^ production in aorta [1]. The inhibition of NADPH oxidase activity may represent a molecular mechanism by which SSR240612 reduces the oxidative stress. This statement is supported by the increased production of O_2_^•−^ by the B_1_R agonist in isolated aorta of glucose-fed rats, whose effect was sensitive to the NADPH oxidase inhibitor apocynin. This observation was confirmed in aorta isolated from glucose-fed rats treated *in vivo* with apocynin prior to the B_1_R agonist. Xanthine oxidase and uncoupling eNOS are unlikely involved in O_2_^•−^ production by the B_1_R agonist since their respective inhibitors (allopurinol and L-NAME) did not affect significantly the pro-oxidative effect of the B_1_R agonist despite they reduced glucose-induced oxidative stress. Thus the inhibition of the oxidative stress by SSR240612 is likely due to the inhibition of the NADPH dependent pro-oxidative effect of B_1_R activation. The inhibition and down-regulation of B_1_R-induced oxidative stress following chronically administered SSR240612 may explain the persistent normalization of high blood pressure, allodynia and insulin resistance in glucose-fed rats.

### Antioxidant defence and SSR240612

It is generally believed that the exposure of cells to oxidative stress is associated with increased antioxidant enzyme activity [47]. Exposure of human endothelial cells to 20 mM glucose for 1–2 weeks increased mRNA expression of CuZnSOD and MnSOD [48]. Likewise, porcine aortic vascular smooth muscle cells cultured in 25 mM glucose for 10 days increased MnSOD mRNA expression [49]. SOD activity was similarly increased in the plasma of 3-week glucose-fed rats [10]. Moreover, SOD and catalase activity were significantly increased during the early stage of diabetes in streptozotocin-treated rats [50]. Thus, the increased expression of MnSOD and catalase in the aorta of 8-week glucose-fed rats is congruent with previous studies and may reflect a compensatory mechanism to the enhanced oxidative stress in glucose-fed rats. It is therefore logical that the reduction of the oxidative stress by chronic treatment with B_1_R antagonist resulted in a normalization of the MnSOD and catalase expression. This further links B_1_R to the generation of the oxidative stress and suggests that the inhibitory effect of SSR240612 on the antioxidant defence is indirect and likely due to the inhibition of the production of ROS.

### Link between B_1_R and angiotensin II (ANG II)

Endogenous ANG II was found to enhance B_1_R expression via AT_1_ receptor in endothelium of small cardiac arteries and cardiomyocytes in two-kidney-one-clip hypertensive rats [51]. Also the experimental model of hypertension induced by chronic infusion of ANG II induced B_1_R expression in rat aorta [52] and spinal cord [53] as previously shown in cultured vascular smooth muscle [54] through a mechanism associated with the oxidative stress and NF-kB. ANG II can activate NF-κB through increases of vascular superoxide production following membrane NADPH oxidase activation [55], [56]. NF-κB is the transcription factor that allows the increased expression of B_1_R [57]. Likewise the model of insulin resistance induced by glucose feeding, the anti-hypertensive effect of SSR240612 was recently demonstrated in ANG II-hypertensive rats and spontaneously hypertensive rats [58].

### Putative role of B_2_R and its relationship with B_1_R expression

Acute treatment with the B_2_R antagonist Icatibant (1 mg/kg) reversed allodynia but not hypertension in rats treated with D-glucose for 12 weeks [59]. Further studies are, however, necessary to determine the influence of B_2_R on the oxidative stress-induced pathological changes in this model. In addition to its pro-nociceptive and pro-inflammatory effects, increasing evidence shows that the B_2_R is nephro- and cardioprotective [60], [61], [62], [63], [64], [65], partly due to nitric oxide (NO) release, and could contribute to the benefit of angiotensin 1-converting enzyme (ACE) inhibitors in models of diabetes and cardiovascular diseases [5], [60], [66], [67].

Whereas the B_1_R is associated with leptin resistance and obesity [13], its beneficial or detrimental role in cardiac ischemia remains conflicting [65], [68], [69], [70] and recently, B_1_R was found implicated in renal fibrosis [71]. The lack of both kinin B_1_R and B_2_R enhances diabetic complications, including nephropathy and neuropathy in Akita diabetic mice [64]. In addition, genetically diabetic mice that lack the B_2_R develop a more severe kidney pathology by age 6 months and develop senescence-associated phenotypes by age 12 months [72], [73]. However, the expression of B_1_R is markedly enhanced in B_2_R knockout mice [72], [74]. Renal expression of B_2_R is also significantly enhanced in B_1_R knockout mice [75], suggesting that the absence of one kinin receptor is compensated by the over-expression of the remaining kinin receptor. Thus, investigation on the respective role of B_1_R and B_2_R in diabetes using genetically modified mice must be cautiously interpreted since it does not simply reflect the absence of a given receptor and may explain apparent contradiction with pharmacological studies. Furthermore, other important genes are affected by genetic deletion of either kinin receptor. For instance, genetic disruption of B_1_R or B_2_R and both receptors decreased ACE and ANG II AT_1_R function and expression in mice abdominal aorta, indicating that kinin receptors regulate AT_1_ receptors and ACE [76], [77]. Moreover, B_2_R knockout mice have increased ANG II AT_2_R mRNA and protein expression that contributes to elevation of NO as compensatory protective mechanism in thrombosis [78]. Finally, our study addressed the role of B_1_R in insulin resistance which corresponds to the early phase of diabetes. The therapy with B_1_R antagonists in a more advanced phase of diabetes as in Akita diabetic mice remains to be clarified.

### Conclusion

The present study provides the first evidence that the B_1_R can perpetuate the oxidative stress by increasing the production of superoxide anion following the activation of NADPH oxidase in a model of insulin resistance. Prolonged inhibition of B_1_R with SSR240612 reversed hypertension, pain polyneuropathy and metabolic alterations in glucose-fed rats. Part of the beneficial effects of SSR240612 appears to be associated with the normalization of B_1_R gene and protein expression which is dependent on the oxidative stress.
